# KidneyNeXt: A Lightweight Convolutional Neural Network for Multi-Class Renal Tumor Classification in Computed Tomography Imaging

**DOI:** 10.3390/jcm14144929

**Published:** 2025-07-11

**Authors:** Gulay Maçin, Fatih Genç, Burak Taşcı, Sengul Dogan, Turker Tuncer

**Affiliations:** 1 Department of Radiology, Beyhekim Training and Research Hospital, Konya 42060, Turkey; gulaymacin@gmail.com; 2 Department of Nephrology, Elazig Fethi Sekin City Hospital, Elazig 23280, Turkey; drfatihgenc@gmail.com; 3 Vocational School of Technical Sciences, Firat University, Elazig 23119, Turkey; 4 Department of Digital Forensics Engineering, Technology Faculty, Firat University, Elazig 23119, Turkey; turkertuncer@firat.edu.tr

**Keywords:** KidneyNeXt, renal tumor classification, computed tomography, convolutional neural network, deep learning, transfer learning, nephrology, medical imaging

## Abstract

**Background**: Renal tumors, encompassing benign, malignant, and normal variants, represent a significant diagnostic challenge in radiology due to their overlapping visual characteristics on computed tomography (CT) scans. Manual interpretation is time consuming and susceptible to inter-observer variability, emphasizing the need for automated, reliable classification systems to support early and accurate diagnosis. **Method and Materials**: We propose KidneyNeXt, a custom convolutional neural network (CNN) architecture designed for the multi-class classification of renal tumors using CT imaging. The model integrates multi-branch convolutional pathways, grouped convolutions, and hierarchical feature extraction blocks to enhance representational capacity. Transfer learning with ImageNet 1K pretraining and fine tuning was employed to improve generalization across diverse datasets. Performance was evaluated on three CT datasets: a clinically curated retrospective dataset (3199 images), the Kaggle CT KIDNEY dataset (12,446 images), and the KAUH: Jordan dataset (7770 images). All images were preprocessed to 224 × 224 resolution without data augmentation and split into training, validation, and test subsets. **Results**: Across all datasets, KidneyNeXt demonstrated outstanding classification performance. On the clinical dataset, the model achieved 99.76% accuracy and a macro-averaged F1 score of 99.71%. On the Kaggle CT KIDNEY dataset, it reached 99.96% accuracy and a 99.94% F1 score. Finally, evaluation on the KAUH dataset yielded 99.74% accuracy and a 99.72% F1 score. The model showed strong robustness against class imbalance and inter-class similarity, with minimal misclassification rates and stable learning dynamics throughout training. **Conclusions**: The KidneyNeXt architecture offers a lightweight yet highly effective solution for the classification of renal tumors from CT images. Its consistently high performance across multiple datasets highlights its potential for real-world clinical deployment as a reliable decision support tool. Future work may explore the integration of clinical metadata and multimodal imaging to further enhance diagnostic precision and interpretability. Additionally, interpretability was addressed using Grad-CAM visualizations, which provided class-specific attention maps to highlight the regions contributing to the model’s predictions.

## 1. Introduction

Kidney cancer refers to a group of malignant neoplasms originating from the renal parenchyma, particularly the proximal tubular epithelium. This disease group presents a heterogeneous clinical spectrum arising from the complex interplay of genetic, environmental, and lifestyle-related factors [1,2]. Renal cell carcinoma (RCC) is the most prevalent malignant subtype, accounting for approximately 85% of all adult kidney tumors. It is typically observed as unilateral, solid, and cortically located masses [3]. Among its histological subtypes, clear cell RCC, papillary, and chromophobe variants are most prominent, each exhibiting distinct clinical behavior, therapeutic responses, and prognostic characteristics [4]. In 2018, more than 400,000 new cases of kidney cancer were reported worldwide, and this number is projected to reach approximately 475,400 annually by 2030 [5,6,7]. The disease is most commonly diagnosed in individuals over 60 years of age, although advancements in imaging technologies have led to increased detection of asymptomatic cases [8]. Major etiological factors include male sex, smoking, hypertension, obesity, and genetic mutations. Furthermore, diets low in fiber and deficient in vitamins have also been identified as potential risk factors [9,10,11,12,13]. Clinically, renal tumors are categorized into three groups: malignant, benign, and normal tissues. Malignant tumors often exhibit an invasive course, requiring surgical or systemic treatment. Benign tumors, such as oncocytoma and angiomyolipoma, are usually noninvasive and become symptomatic only in certain cases [14,15]. On the other hand, normal tissues or simple cysts are considered physiological structures that do not necessitate medical intervention. From a clinical management perspective, reliable and early differentiation among these three categories is crucial for devising patient-specific treatment strategies and avoiding unnecessary invasive procedures. Currently, imaging techniques, particularly computed tomography (CT), are widely utilized for evaluating renal tumors. However, interpreting these images relies heavily on the clinician’s experience, and variability between observers may result in diagnostic ambiguity. To address these limitations, artificial intelligence (AI)-driven methods have emerged as promising tools to streamline image analysis and enhance diagnostic reliability.

In this study, we introduce a convolutional neural network (CNN)-based [16] classification model developed using CT images. The model aims to differentiate malignant, benign, and normal kidney structures, offering a supportive tool for more consistent and data-driven clinical assessments.

### 1.1. Literature Review

Over the past decade, numerous deep-learning-based approaches have been proposed for the classification and detection of kidney tumors using medical imaging data, particularly CT and magnetic resonance imaging (MRI). These studies have explored various neural network architectures, data preprocessing techniques, and fusion strategies to improve diagnostic accuracy. Below, we present a summary of key contributions in this field, highlighting their methodologies, datasets, performance outcomes, and limitations.

Abdelrahman et al. [17] aimed to classify kidney cancer CT images using a combination of EfficientNet and U Net architectures. Their study utilized the KiTS19 dataset, which comprises 210 CT volumes totaling 7899 images. A major limitation was the high computational cost due to the large number of model parameters. Among the tested variants, EfficientNet B4 and EfficientNet B7 achieved the classification performance with a mean Intersection over Union (IoU) accuracy of 98.0%. Bolocan et al. [18] developed a CNN to detect and classify clear cell renal cell carcinoma (ccRCC) based on multiphase CT images. The training dataset consisted of 457 healthy right kidneys, 456 healthy left kidneys, and 76 pathological right and 84 pathological left kidneys. The study’s main drawback was the relatively small dataset and the time-consuming manual preprocessing steps. The model achieved an accuracy of 88.5% in tumor classification. Ghosh et al. [19] proposed a system integrating fuzzy-logic-based image enhancement, a dual transfer learning network, and a weighted ensemble machine learning classifier for kidney tumor detection in CT images. The dataset included 8260 CT images (5977 normal and 2283 tumorous) collected from hospitals in Bangladesh and was augmented to 12,000 balanced images. Variability in image quality was noted as a significant limitation. The model, termed streamliners, achieved results with 99.25% accuracy, 99.30% recall, 99.27% precision, and a 99.28% F1 score. Anush et al. [20] developed a deep-learning-based ensemble approach for fully automated detection of small renal masses (SRMs) using contrast-enhanced MRI (CE MRI). Their dataset included 25,025 slices from 118 patients, covering various solid renal tumor subtypes. A key limitation was the lack of multiparametric MRI usage, despite the inclusion of data from multiple MRI systems. The model yielded a Dice score of 91.2% for kidney segmentation, 86.2% recall, 83.3% precision, and an 84.7% F1 score for tumor detection. Kittipongdaja et al. [21] developed two models, namely 2.5D ResUNet and 2.5D DenseUNet, for automatic kidney segmentation in CT images, aimed at analyzing the malignancy potential of complex renal cysts. The training and validation datasets were derived from KiTS19 (210 and 60 patients, respectively), while generalizability was tested on four Thai patients. The limited number of Thai patients posed a challenge for statistical representativeness. The 2.5D DenseUNet achieved the performance with a Dice score of 95.82% on the KiTS19 validation set and 87.60% on the Thai patient data. Magherini et al. [22] designed a fully automated classification tool to distinguish ccRCC from oncocytoma using radiomic and deep features extracted from CT images. Data from 61 patients at Careggi University Hospital (29 ccRCC and 34 oncocytoma) and 271 patients from the KiTS2019 dataset were used. The primary limitation was the class imbalance between benign and malignant cases. The performing model reported 86.84% accuracy, 94.59% recall, 52.94% specificity, and a balanced accuracy of 73.77%. Pavarut et al. [23] sought to improve classification performance by utilizing multimodal medical images (CT and MRI) and data enrichment through partially synthesized missing modalities using Conditional CycleGAN. The dataset from Tokyo Medical and Dental University contained two classes angiomyolipoma (AML) and ccRCC across five imaging modalities (3 CT, 2 MRI). A critical limitation was potential misalignment between modalities. The proposed early/intermediate feature level fusion method achieved an AUC of 94.5%, demonstrating improved classification performance. Khan et al. [24] explored deep transfer learning using fine-tuned EfficientNetV2 and ConvNeXt models for accurate classification of chronic kidney diseases in CT images. The study utilized the “CT Kidney” dataset, comprising 12,446 images across four classes (normal, cyst, stone, tumor). A notable limitation was the requirement for trimming to address class imbalance. The fine-tuned EfficientNetV2B0 model delivered performance with 99.75% accuracy, 99.63% recall, 99.75% precision, and a 99.75% F1 score. Patel et al. [25] introduced an innovative deep learning framework for kidney tumor detection and classification using CT images, combining 3D Adaptive Trans ResUNet (3D ATR) and Multiscale Trans Residual Attention Network (MT RAN). The study employed KiTS19 2 (200 images) and KiTS21 datasets. A major limitation was the high computational demand and parameter intensity of the Trans ResUNet architecture. The proposed framework achieved 96.18% accuracy, 95.39% recall, 86.72% precision, and a 90.85% F1 score. Zhao et al. [26] proposed a method combining 3D U Net and ResNet architectures for automatic detection and classification of renal masses in CT images. The dataset comprised 610 CT series from 490 patients. A significant limitation was the reduced model performance for small (<5 mm) lesions and low-contrast solid masses. The classification model achieved 90.56% accuracy overall, 86.05% accuracy for lesions < 5 mm, and 91.97% for lesions ≥ 5 mm. For solid masses, the model yielded 94.74% precision, 83.72% recall, and an F1 score of 89%.

Compared to the aforementioned studies, the proposed KidneyNeXt model provides several key advantages. Many prior approaches utilized computationally intensive architectures such as three-dimensional networks, ensemble methods, or transformer-based systems. In contrast, KidneyNeXt is designed as a lightweight and high-performing convolutional neural network with significantly fewer parameters and faster inference capability. Additionally, while previous studies often relied on single-center or limited datasets, our model was evaluated using three distinct sources, including a custom-collected dataset, thereby demonstrating strong generalization across diverse data. Furthermore, unlike studies that focused solely on segmentation or binary classification tasks, KidneyNeXt achieves multi-class tumor classification with exceptionally high accuracy. The study also integrates interpretability analysis using Grad-CAM and includes misclassification examples, contributing to transparency and clinical trust. These aspects establish KidneyNeXt as a practical, efficient, and generalizable solution for renal tumor classification using CT images.

### 1.2. Motivation and Our Model

In recent years, deep-learning-based models have demonstrated remarkable success in medical image classification and have provided effective solutions to a wide range of clinical problems. High-resolution imaging techniques such as CT have become particularly valuable in the detection and classification of kidney tumors. However, the inherently high dimensional and heterogeneous nature of CT data poses challenges for manual interpretation, often leading to time dependent errors and inter-observer variability. Consequently, there is a growing need for efficient, generalizable, and parameter optimized artificial intelligence solutions that can be integrated into clinical decision support systems. Motivated by these challenges, we propose a novel CNN architecture named KidneyNeXt for the classification of kidney tumors into benign, malignant, and normal categories. The design of KidneyNeXt incorporates a balanced depth scale strategy by combining multi-branch convolutional pathways, group convolutions, hierarchical building blocks, and global feature extraction mechanisms. Furthermore, the model is trained using a protocol that leverages both transfer learning and fine-tuning techniques, allowing it to generalize effectively across datasets collected from multiple sources.

### 1.3. Novelties and Contributions

The KidneyNeXt model developed in this study introduces several novel aspects and valuable contributions to the existing body of literature, which can be summarized as follows:

- KidneyNeXt presents a novel CNN architecture that integrates parallel convolutional pathways, group convolutions, and modules capable of extracting multiscale features. This design enables the model to effectively capture both fine grained local features and global structural information.
- The model is trained and tested on three distinct datasets collected from geographically and clinically diverse sources. This strategy enhances the model’s robustness against inter-institutional variability and improves its generalizability in real-world clinical settings.
- Initialized with ImageNet 1K pre-trained weights, the model benefits from transfer learning to achieve high accuracy even with a relatively limited number of CT images. Despite its strong performance, the architecture remains lightweight, comprising approximately 7.1 million parameters, which supports faster inference and reduced computational burden.
- The model achieved high accuracy, F1 score, and recall across benign, malignant, and normal kidney tissue classes, outperforming several existing models reported in the literature.
- The balance between accuracy and computational efficiency demonstrated by KidneyNeXt suggests its potential utility as a semi-automated decision support tool in clinical workflows.

Taken together, this work not only introduces a novel deep learning architecture but also provides a clinically relevant classification model with strong generalization capabilities, thereby making a meaningful contribution to the field of AI-assisted medical diagnosis.

## 2. Materials and Methods

In this study, a comprehensive experimental design was established to develop and evaluate a CNN-based model for the classification of kidney tumors. To enhance the model’s robustness and generalizability, multiple datasets obtained from different sources were utilized. The integration of datasets with diverse imaging characteristics helps reduce dataset-specific learning bias and enables the model to capture a broader spectrum of tumor-related patterns. This multi-source data approach not only strengthens the clinical relevance of the results but also enhances the model’s potential for real-world diagnostic applications. These datasets differ in terms of acquisition protocols, image quality, and demographic characteristics, allowing us to evaluate the generalization ability of the proposed model across heterogeneous sources.

### 2.1. Collected Dataset

**Figure 1:**
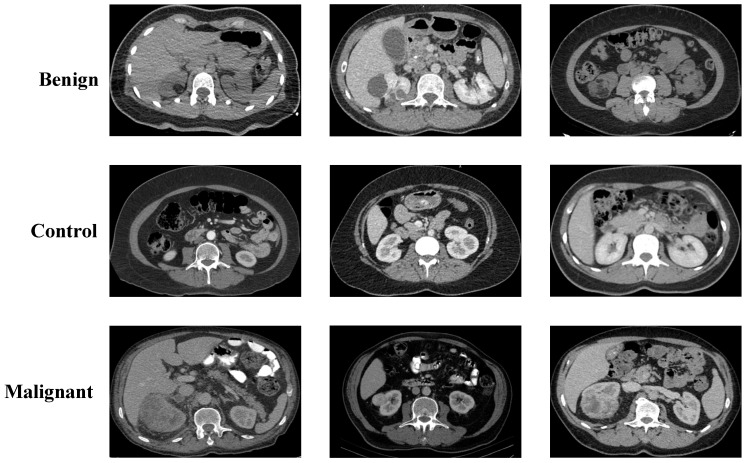
Sample axial CT images representing benign, control, and malignant kidney cases from the collected dataset.

The first dataset used in this study was retrospectively collected by our research team. All images were obtained from the archive records in accordance with an approved protocol by the Elazığ Fethi Sekin City Hospital Clinical Research Ethics Committee (Session No: 2025/6 20, 20 March 2025). The dataset consists solely of CT scans, which were cropped appropriately and anonymized by removing all patient identifiers. Final images were saved in .png format. Classification of the images was performed independently by an experienced nephrologist and radiologist, resulting in three categories: malignant, benign, and control. A total of 1147 malignant images were included, with a mean patient age of 60.23 ± 7.95 years (range: 53–76). The benign group comprised 1919 images, with a mean age of 65.27 ± 5.22 years (range: 58–77). The control group included 1133 images, and the mean age was calculated as 60.18 ± 6.25 years (range: 55–78). Representative axial CT images for each category are presented in Figure 1.

### 2.2. Kaggle CT KIDNEY Dataset

**Figure 2:**
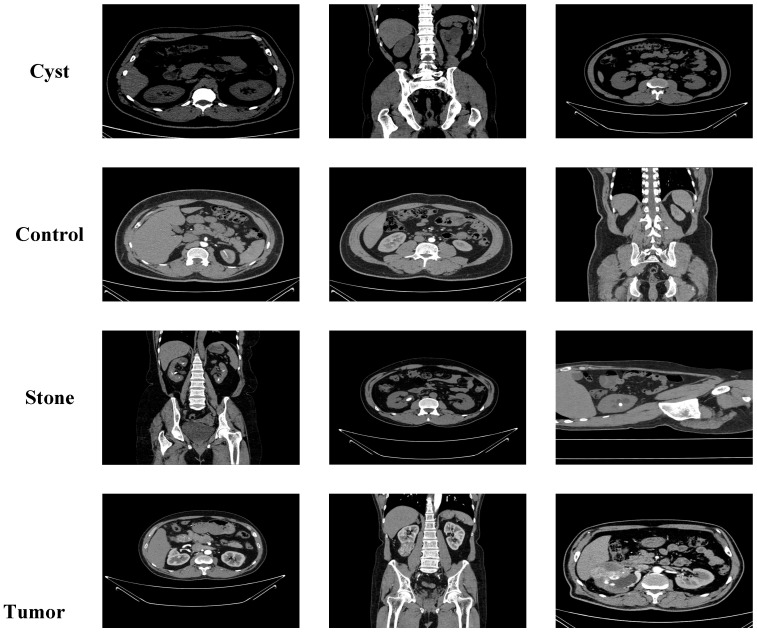
Sample images from the Kaggle CT KIDNEY Dataset.

The second dataset used in this study is the publicly available CT KIDNEY DATASET: Normal Cyst Tumor and Stone, hosted on the Kaggle platform [27]. The images were collected from the Picture Archiving and Communication System (PACS) of several hospitals in Dhaka, Bangladesh. Each image belongs to a patient already diagnosed with either kidney tumor, cyst, normal kidney structure, or kidney stones. Both coronal and axial sections, from contrast-enhanced and non-contrast CT studies, were selected in accordance with whole abdomen and urogram imaging protocols. DICOM format scans were carefully curated for each diagnostic category, segmented to include relevant anatomical regions, and anonymized by removing patient information and metadata. The images were then converted to .jpg format using a lossless method. Following this, each image was independently verified by a radiologist and a medical technologist to ensure quality. This dataset comprises a total of 12,446 images, distributed as follows: 3709 cyst images, 5077 normal images, 1377 stone images, and 2283 tumor images. Its rich and diverse structure supports the model’s ability to distinguish among various types of kidney lesions. Representative examples are shown in Figure 2.

### 2.3. KAUH: Jordan Dataset

**Figure 3:**
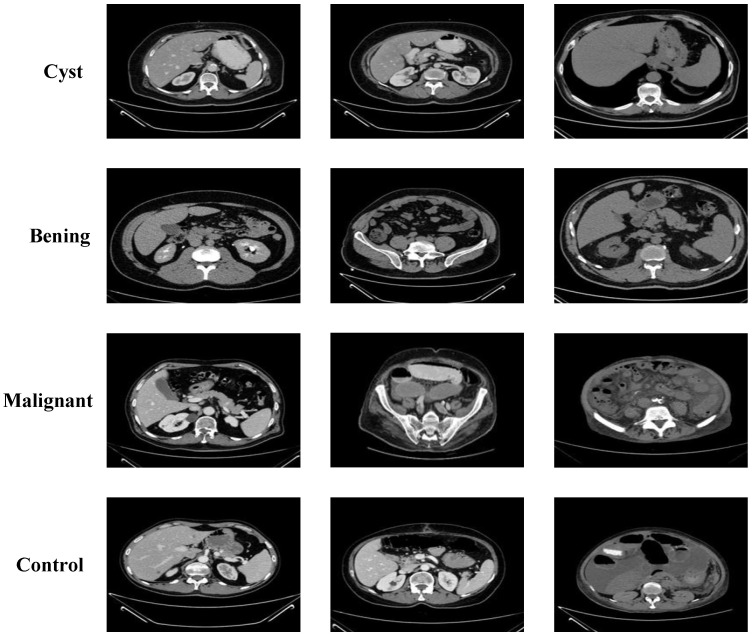
Sample images from the KAUH: Jordan Dataset.

The third dataset was obtained from King Abdullah University Hospital (KAUH), Jordan, and includes CT images of various kidney conditions [28]. The dataset is categorized into four groups: benign, malignant, normal case with cyst, and normal (control). Each image underwent diagnostic validation to ensure accuracy. The dataset contains a total of 7770 images. The benign group includes 2660 images, with a mean patient age of 60.35 ± 8.92 years (range: 32–80). The malignant group comprises 1540 images, with an average age of 61.75 ± 11.23 years (range: 38–85). The normal case with cyst group contains 1330 images, with a mean age of 62.84 ± 13.17 years (range: 36–88). The control group consists of 2240 images, and the average age is 52.47 ± 14.21 years (range: 20–77). Sample images representing each category are displayed in Figure 3.

### 2.4. The Proposed KidneyNeXt

In this study, a deep-learning-based model was designed and a comprehensive experimental protocol was implemented for the classification of kidney tumors using CT images. The methodological pipeline comprises dataset compilation, image preprocessing, and the application of a customized CNN architecture referred to as KidneyNeXt. To ensure the reliability and generalizability of the model’s performance, a multi-source dataset strategy was adopted. The training process was supported by both transfer learning and fine-tuning strategies. This section systematically outlines each stage of the proposed approach.

**Step 1:** To facilitate robust tumor classification, three distinct CT datasets were combined in this study. Details regarding these datasets are provided in Section 2.1, Section 2.2 and Section 2.3. All data were anonymized in accordance with ethical guidelines.

**Step 2:** The images were converted to .png or .jpg format and then resized to 224 × 224 pixels. Each image was represented in RGB color channels. The dataset includes both axial and coronal planes and encompasses contrast-enhanced and non-contrast CT scans.

**Step 3:** No data augmentation techniques were applied. The combined dataset was randomly divided into 80% for training and 20% for testing. Additionally, 30% of the training data were reserved for validation.

**Step 4:** KidneyNeXt is a hierarchically structured CNN architecture featuring multi-path processing blocks and residual connections (see Table 1). The input image $F_{\mathrm{base}\;}\in R^{56\times 56\times 96}$ is processed through two parallel convolutional layers:

(1) $$F_{1}=BN\left(GELU\left({Conv}_{4\times 4}(I)\right)\right),F_{2}=BN\left(GELU\left({Conv}_{4\times 4}(I)\right)\right)$$

The average of these two paths forms the base feature map $F_{\mathrm{base}\;}\in R^{56\times 56\times 96}$, which is subsequently forwarded through four parallel processing streams: max pooling $P_{\max\;}$, average pooling $P_{\mathrm{avg},\;}$, and two separate group convolutions $G_{1},G_{2}$. The outputs of these branches are concatenated, followed by channel compression:

(2) $$F_{\mathrm{concat}\;}=Concat\left(P_{max},P_{\mathrm{avg}\;},G_{1},G_{2}\right)\in R^{56\times 56\times 384}$$

(3) $$F_{\mathrm{compressed}\;}={Conv}_{1\times 1}\left(F_{\mathrm{concat}\;}\right)\in R^{56\times 56\times 96}$$

These blocks are repeated in a hierarchical manner across four stages, with output channel sizes of 96, 192, 384, and 768, respectively. The final tensor $F_{\mathrm{final}\;}\in R^{7\times 7\times 768}$ is transformed into a 768 dimensional feature vector using global average pooling (GAP):

(4) $$f=GAP\left(F_{\mathrm{final}\;}\right)$$

For the final classification, the feature vector is passed through a fully connected layer followed by a softmax function:

(5) $$\hat{y}=Softmax(W\cdot f+b)$$

**Figure 4:**
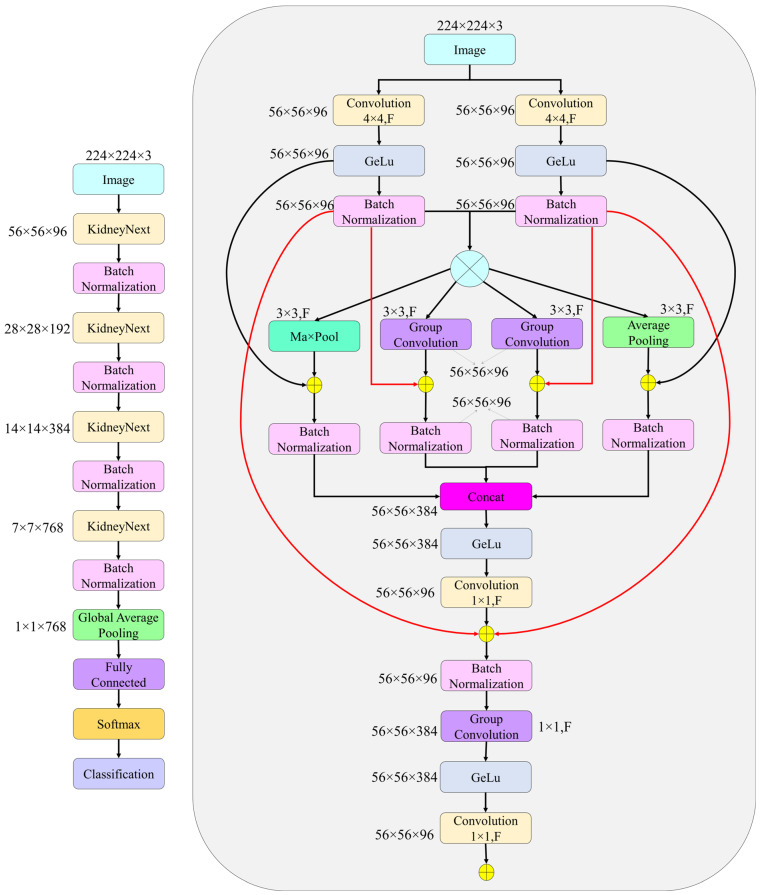
The overall architecture of the proposed KidneyNeXt model.

The architecture of the proposed model is illustrated in detail in Figure 4. The network begins by applying two distinct convolutional operations to the input image, extracting fundamental features that are subsequently refined through progressively deeper representations within the multi-path processing KidneyNeXt blocks. Each block simultaneously integrates diverse pooling strategies and group convolutions, thereby preserving both spatial and structural variability in the feature maps. Residual connections and compressed transition layers are incorporated between stages, enhancing the model’s learning capacity while mitigating the risk of overfitting. In the final stage, global average pooling followed by a fully connected layer is used to compute the class scores. With a total of approximately 7.1 million trainable parameters, the architecture achieves a favorable balance between compactness and high classification performance.

**Step 5:** The model was initially pre-trained on the ImageNet 1K dataset. During this stage, training was performed for 90 epochs using the AdamW optimization algorithm, with a learning rate set to 1 × 10^−3^ and a weight decay coefficient of 1 × 10^−4^. Following pre-training, the model was fine-tuned on the combined dataset consisting of kidney CT images. The dataset was split into 80% for training and 20% for testing, with 30% of the training data further allocated for validation. The final training stage employed the Stochastic Gradient Descent with Momentum (SGDM) optimizer, using a momentum value of 0.9, over 30 epochs. The initial learning rate was set to 0.01, with a batch size of 128, and an L2 regularization coefficient of 1 × 10^−4^. Data shuffling was applied at the beginning of each epoch. Neither checkpointing nor early stopping mechanisms were used during training. All training processes were conducted in an automated hardware environment.

## 3. Experimental Results

All experimental procedures were conducted on a workstation running Windows 11 operating system. The hardware configuration included a 13th generation Intel^®^ Core™ i9 13900K processor (Santa Clara, CA, USA), 128 GB of RAM, 1 TB SSD storage, and an NVIDIA (Santa Clara, CA, USA) GeForce RTX 4080 Super GPU. Model design, preprocessing steps, and all training and staged validation procedures were implemented using the MATLAB 2023b software environment.

### 3.1. Performance Evaluation on Collected Dataset

The initial evaluation of the model was conducted using the Collected Dataset, which was retrospectively obtained from the archives of Elazığ Fethi Sekin City Hospital. This dataset comprises three categories malignant, benign, and control with all images independently labeled by an experienced nephrologist and radiologist. The KidneyNeXt model was trained using an 80% training/20% testing split, with 30% of the training data further reserved for validation. Table 2 summarizes the class-wise distribution of images used during the training and testing phases for the Collected Dataset. The dataset is evenly divided across the three categories, ensuring a balanced representation in both the training and test sets. This structure provides a robust foundation for the reliable evaluation of the KidneyNeXt model.

**Figure 5:**
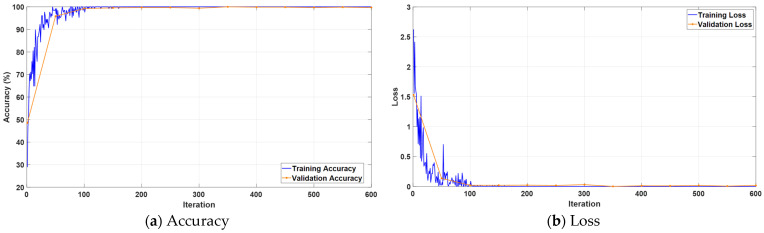
Training curves of the KidneyNeXt model showing (**a**) accuracy and (**b**) loss over iterations.

Figure 5 illustrates the progression of accuracy and loss metrics over the course of training iterations for the proposed KidneyNeXt model. As shown in Figure 5a, both training and validation accuracy exhibit a rapid increase from the early iterations, surpassing 95% within a short span and maintaining consistently high levels throughout the training process. Figure 5b demonstrates that the training and validation losses converge quickly to minimal values. The observed stability in both accuracy and loss curves indicates that the model successfully avoids overfitting and possesses strong generalization capabilities. These results confirm that the KidneyNeXt model not only exhibits an effective learning dynamic but also delivers robust classification performance.

**Figure 6:**
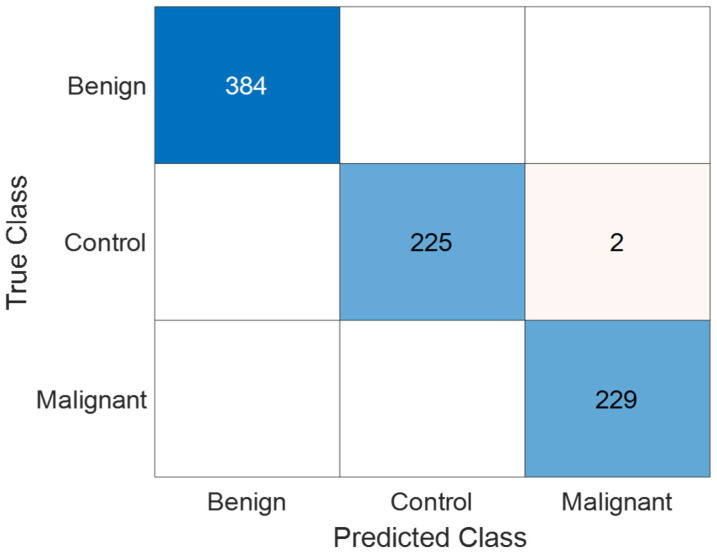
Confusion matrix of the KidneyNeXt model showing classification results on the test set of the Collected Dataset.

Figure 6 presents the confusion matrix illustrating the classification performance of the KidneyNeXt model on the Collected Dataset. The matrix demonstrates that the model accurately distinguishes among all three classes benign, control, and malignant. The most notable misclassification occurred when two instances from the control class were incorrectly labeled as malignant. Apart from this, no significant confusion was observed between other classes. These results indicate that the model is capable of achieving high inter-class discrimination accuracy.

Table 3 presents the classification performance of the KidneyNeXt model on the test set, including metrics such as True Positives (TP), True Negatives (TN), False Positives (FP), and False Negatives (FN), along with the computed precision, recall, F1 score, and accuracy for each class. The model achieved its highest performance in the benign class, with a perfect 100% accuracy. For both the control and malignant classes, F1 scores exceeded 99%. When evaluating the overall performance, the macro-averaged metrics reached 99.71%, confirming that the model not only exhibits high sensitivity but also maintains a well-balanced performance across all classes.

**Figure 7:**
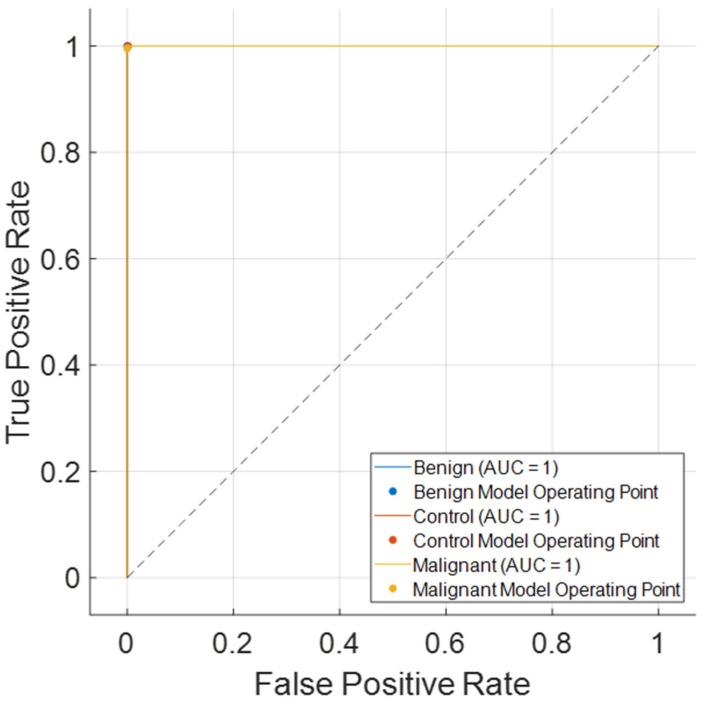
ROC curves for benign, control, and malignant classes on the test set of the Collected Dataset.

Figure 7 shows the Receiver Operating Characteristic (ROC) curves for the three classes: benign, control, and malignant. All classes achieved an Area Under the Curve (AUC) of 1.00, indicating perfect class separability and excellent discriminatory ability of the KidneyNeXt model. This result reinforces the model’s robustness and highlights its effectiveness in correctly identifying each tumor type without overlap. The near-perfect ROC performance complements the confusion matrix (Figure 6), where misclassifications were minimal and class-wise performance was balanced.

**Figure 8:**
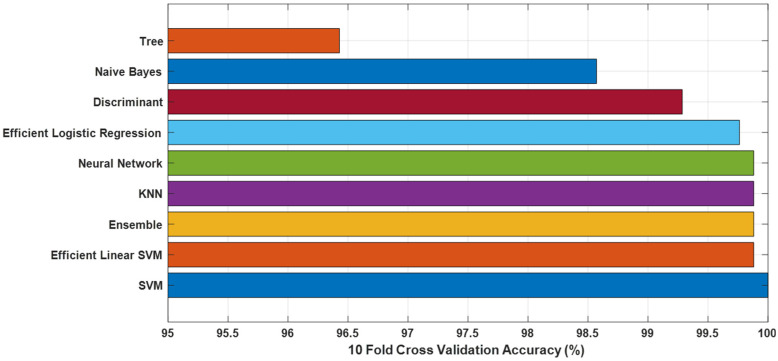
Ten-fold cross-validation accuracy of different traditional classifiers using deep features extracted from FC Layer of the KidneyNeXt model on the test set of the Collected Dataset.

To further evaluate the generalization performance of the KidneyNeXt model and address concerns related to potential overfitting, we extracted deep features from the final fully connected layer of the trained network using the test partition of the Collected Dataset. These features were subsequently used to train and test multiple traditional machine learning classifiers under a 10-fold cross-validation framework (see Figure 8). A number of features were extracted from each image and labeled accordingly. The extracted features were then used as input to various classification algorithms, including Support Vector Machines (SVM) [29,30], K-Nearest Neighbors (KNN) [31], Neural Networks [32], Decision Trees [33], Naive Bayes [34], and Ensemble methods. Among these, the standard SVM classifier yielded the highest accuracy of 100.00%, followed closely by Efficient Linear SVM, Ensemble, KNN, and Neural Network classifiers, all achieving accuracies above 99.80%. These results underscore the high discriminative power of the features learned by KidneyNeXt and substantiate its robustness and generalizability across traditional machine learning paradigms.

**Figure 9:**
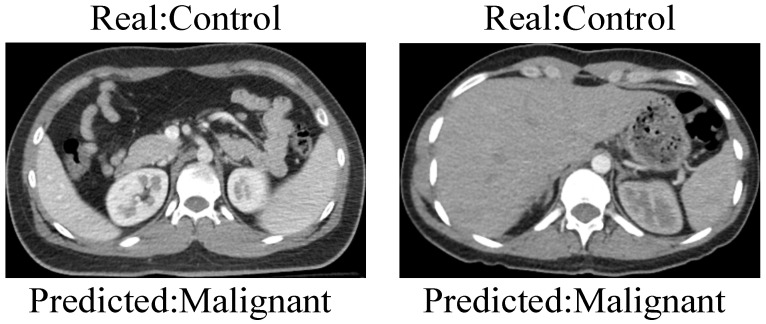
Examples of misclassified control cases that were incorrectly predicted as malignant by the KidneyNeXt model. These samples correspond to the two misclassified instances shown in Figure 6.

Although the KidneyNeXt model achieved high classification performance, as reflected in the confusion matrix shown in Figure 6, a small number of misclassifications was observed. Specifically, two control images were incorrectly classified as malignant. Figure 9 illustrates examples of these misclassified control cases. Visual ambiguity or subtle texture similarities to pathological structures may have contributed to these errors. Such instances emphasize the importance of incorporating uncertainty estimation and explainability tools to improve clinical reliability.

### 3.2. Performance Evaluation on Kaggle CT KIDNEY Dataset

The second phase of experimental validation for the KidneyNeXt model was conducted using the publicly available Kaggle CT KIDNEY dataset. This dataset comprises CT images categorized into four diagnostic classes: cyst, normal kidney, stone, and tumor. The large number of samples and the diversity among classes provided a suitable testing environment to assess the generalizability of the model.

Table 4 presents the distribution of training and test images for each class. This balanced allocation is essential for a fair assessment of the KidneyNeXt model’s class-wise performance. A sufficient number of training samples enabled the model to effectively learn generalizable patterns, while an adequate number of test instances improved the statistical reliability of the performance evaluation.

**Figure 10:**
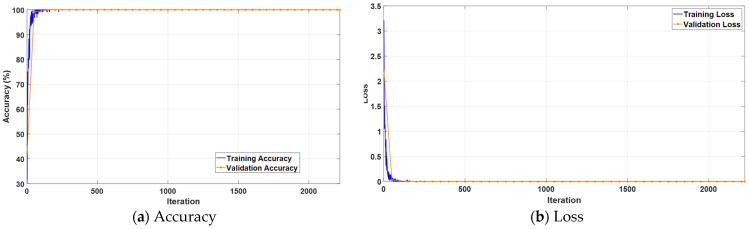
Training curves of the KidneyNeXt model on the Kaggle CT KIDNEY dataset showing (**a**) accuracy and (**b**) loss over iterations.

Figure 10 illustrates the variation in accuracy and loss values across training iterations. The accuracy curves in the left panel indicate that the model achieved high and stable accuracy levels on both the training and validation sets within a short number of iterations. The loss curves in the right panel demonstrate that the training process progressed efficiently and consistently, with a minimal risk of overfitting. These findings highlight the model’s strong learning capacity and generalization ability.

**Figure 11:**
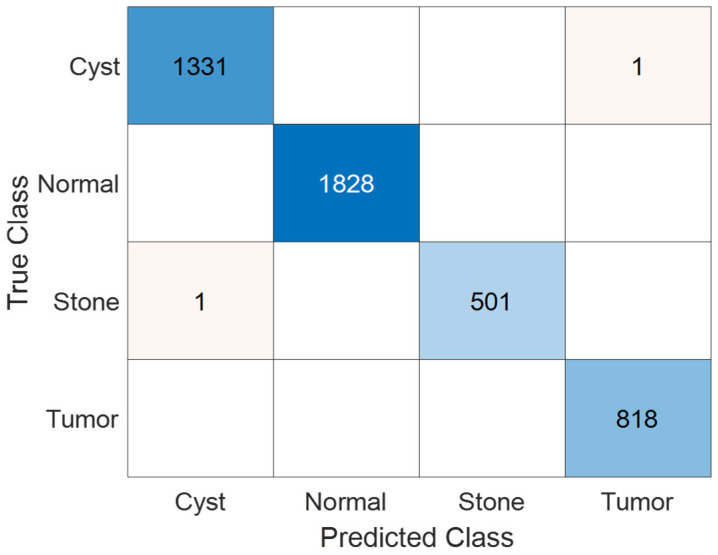
Confusion matrix showing the classification results of the KidneyNeXt model on the test set of the Kaggle CT KIDNEY Dataset.

The confusion matrix in Figure 11 provides a detailed visualization of the model’s classification performance on the test set. The KidneyNeXt model accurately predicted the vast majority of instances across all four classes. Only a very small number of misclassifications were observed in the cyst and stone categories. Remarkably, the normal and tumor classes were classified with near perfect accuracy, clearly highlighting the strong class discrimination capability of the KidneyNeXt architecture.

Table 5 presents the class-wise True Positive (TP), False Positive (FP), False Negative (FN), and True Negative (TN) values, along with the calculated recall, precision, F1 score, and accuracy metrics for each category. The KidneyNeXt model achieved over 99% performance across all four classes, with an overall accuracy of 99.96%. Notably, the model attained 100% recall and F1 score for the normal class, underscoring its excellent detection capability in that category. The macro-average values reported in the “Overall” row indicate that the model performs consistently across all classes, without significant performance variance.

### 3.3. Performance Evaluation on KAUH: Jordan Dataset

The third experimental evaluation of the model was conducted using the KAUH: Jordan Dataset, which consists of CT images provided by King Abdullah University Hospital (KAUH). This dataset includes images corresponding to four distinct kidney conditions: benign, malignant, cyst, and normal, and is characterized by its diversity, reflecting real-world clinical scenarios. The KidneyNeXt model was trained and rigorously validated on this dataset, with performance analyzed in detail across both validation and test subsets.

Table 6 presents the numerical distribution of training and test images for each class. The training data exhibit a balanced structure in which all classes are adequately represented, and a similar balance is maintained in the test set. This structure provides a suitable sample for accurately assessing both the learning performance and generalization capability of the model.

**Figure 12:**
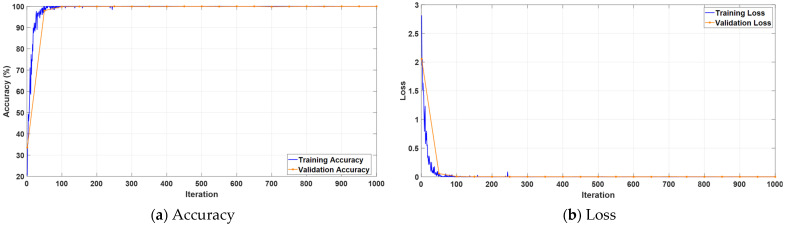
Training curves of the KidneyNeXt model on the KAUH: Jordan Dataset showing (**a**) accuracy and (**b**) loss over iterations.

Figure 12 illustrates the accuracy and loss trajectories of the KidneyNeXt model during training with the KAUH: Jordan Dataset. In panel a, the accuracy curves rise rapidly from the early iterations and stabilize after approximately 100 iterations. In panel b, both training and validation loss curves reach their minimum values quickly, indicating that the model was optimized in a stable manner without signs of overfitting.

**Figure 13:**
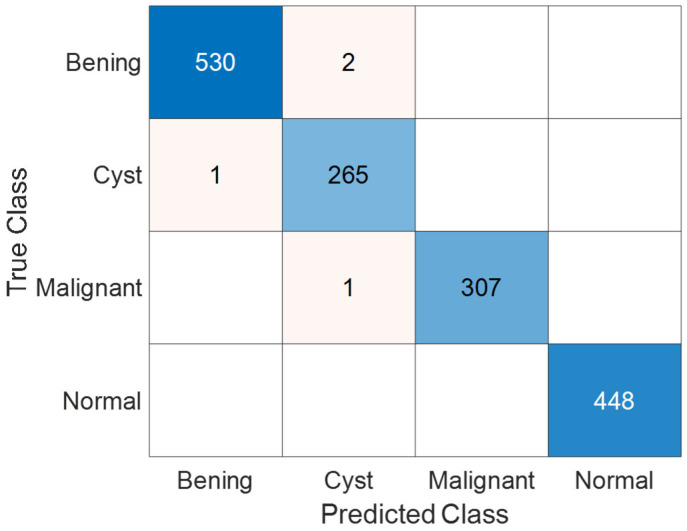
Confusion matrix showing the classification results of the KidneyNeXt model on the test set of the KAUH: Jordan Dataset.

Figure 13 presents the confusion matrix depicting the test performance of the KidneyNeXt model on the KAUH: Jordan Dataset. The matrix provides a detailed visualization of correct and incorrect classifications across all categories. According to the results, the model achieved its highest accuracy in the normal and malignant classes, while only a very limited number of misclassifications occurred in the benign and cyst categories. These outcomes suggest that the model was largely successful in accurately distinguishing between all four classes.

Table 7 reports the class-wise metrics of the KidneyNeXt model on the KAUH test dataset, including True Positive (TP), True Negative (TN), False Positive (FP), and False Negative (FN) values, along with performance indicators such as accuracy, recall, precision, and F1 score. The overall accuracy of the model was calculated as 99.74%, while the macro-averaged recall, precision, and F1 score were 99.73%, 99.72%, and 99.74%, respectively. These results indicate that the model not only delivers high performance but also maintains balanced and consistent classification capabilities across all classes. The experimental analyses clearly demonstrate that the KidneyNeXt model can perform accurate and consistent classification across diverse CT datasets. In all three datasets evaluated, the model successfully distinguished between benign, malignant, cyst, and normal classes with high recall, precision, and F1 score. Each dataset exhibited low error rates, strong generalization ability, and balanced class level performance. Notably, the model maintained its stability across multi-source testing scenarios, offering promising evidence for its applicability in real-world clinical settings. In this regard, integrating the proposed model into medical image-based diagnostic systems may provide valuable support for early diagnosis processes.

## 4. Discussion

In this study, a customized CNN architecture, KidneyNeXt, was developed for the automated classification of kidney tumors using CT images and was extensively evaluated across multiple datasets. The experimental results demonstrated that the proposed model accurately distinguishes between benign, malignant, and normal classes. Owing to its parametric efficiency and transfer learning capability, the model maintained strong classification performance even under limited data conditions. These findings suggest that the KidneyNeXt architecture offers a promising framework that can be integrated into clinical decision support systems to assist in the early diagnosis of kidney tumors. As summarized in Table 8, state of the art models such as those proposed by Khan et al. (2025) and Loganathan et al. (2025) [35,36] demonstrate strong classification performance on kidney CT datasets. However, issues like class imbalance and inter-class variability remain critical challenges for generalization.

Numerous deep-learning-based approaches have been proposed in the literature for the classification of kidney tumors using CT images. For instance, Khan et al. (2025) reported a high accuracy of 99.30% with their ConvLSTM and Inception-based hybrid model; however, this performance dropped to 91.31% when applied to a four class dataset [35]. Similarly, Loganathan et al. (2025) achieved an accuracy of 98.87% with their EACWNet architecture, though their model exhibited low precision in the stone class [36]. Rehman et al. (2025) obtained 99.20% accuracy using a combination of Swin ViT and DeepLabV3+, but the high computational complexity was noted as a major limitation [37]. Ensemble-based methods such as those proposed by Ayogu et al. (2025) [40], and EfficientNet-based models introduced by Hossain et al. (2025) [44], also faced challenges including class imbalance and low recall for certain categories. In contrast, the proposed KidneyNeXt model significantly overcomes these limitations, consistently achieving over 99.70% in accuracy, precision, recall, and F1 score across three distinct datasets. Despite differences in data origin and characteristics, the KidneyNeXt model maintained consistently high performance, indicating strong generalizability and robustness across diverse imaging environments. Its architecture, which integrates parallel convolutional pathways and group convolutions, combined with training on multi-source data, enhances both generalizability and inter-class discrimination. In this regard, KidneyNeXt demonstrates superior performance over existing approaches and stands out as a reliable and clinically applicable AI model for kidney tumor classification. Unlike many previous approaches relying on parameter-heavy ensemble methods, transformers, or 3D architectures, KidneyNeXt employs a streamlined architecture with parallel convolutional and group convolution blocks, enabling efficient and discriminative feature learning while maintaining low computational complexity.

**Figure 14:**
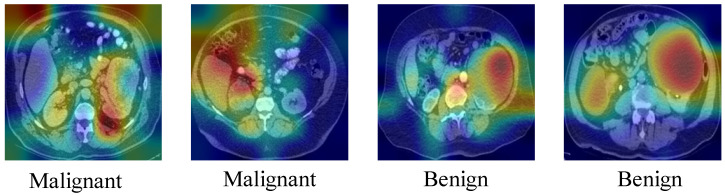
Grad-CAM visualizations of malignant and benign cases from the Collected Dataset.

To enhance interpretability, Gradient-weighted Class Activation Mapping (Grad-CAM) [50] was used to highlight the discriminative regions that contributed to the KidneyNeXt model’s predictions (see Figure 14). The resulting heatmaps demonstrate that the model focused on clinically relevant anatomical areas, such as zones with altered tissue density or mass-like formations. In malignant cases, the activated regions appeared broader and asymmetrical, consistent with tumoral spread, while in benign cases, attention was more localized and well-defined. These visualizations offer insights into the model’s decision-making process and help support its clinical reliability.

Limitations:

- The datasets employed in this study were not sourced from a multi-center framework, which may limit the diversity of the study population. This lack of multi-institutional representation could introduce population bias and affect the model’s generalizability to broader clinical settings.
- The current approach relies exclusively on imaging data. Important clinical variables such as sex, body mass index (BMI), and renal function indicators were not included, which may constrain the model’s ability to account for inter-individual variability in real-world diagnostic settings.

Future Directions:

- Future research efforts will aim to evaluate the generalizability of the proposed model using external datasets collected from institutions across varied geographic locations and population groups. Emphasis will be placed on including cohorts that represent diverse ethnic, demographic, and clinical profiles to ensure robustness and fairness across different real-world scenarios.
- Upcoming work will also aim to incorporate structured clinical metadata, including demographic and laboratory information, into the classification pipeline. This integration is expected to improve the model’s interpretability, clinical relevance, and overall diagnostic robustness.
- Future research will explore the practical integration of the KidneyNeXt model into clinical workflows, including inference time analysis, PACS compatibility, and system interoperability. Pilot studies and usability evaluations in real clinical settings will be conducted to assess feasibility and acceptance.

## 5. Conclusions

This study presents KidneyNeXt, a tailored CNN designed to classify kidney tumors using CT images. By leveraging a hybrid architectural design featuring parallel convolutional pathways, grouped convolutions, and hierarchical feature extraction blocks the model effectively captures diverse spatial features associated with renal lesions. The training strategy combined transfer learning and fine tuning over a multi-source CT dataset, thereby enhancing both robustness and generalizability. Experimental evaluations across three publicly available datasets consistently demonstrated that KidneyNeXt achieves performance compared to existing deep-learning-based classifiers. The model excelled in distinguishing between normal kidney tissue and various tumor types, including benign, cystic, and malignant lesions, even under challenging conditions, such as class imbalance and subtle inter-class variations. While the results are promising, several directions remain for future research. Incorporating clinical metadata could provide additional context to improve classification accuracy and facilitate personalized diagnosis. Furthermore, expanding the model to incorporate multimodal imaging could offer more comprehensive diagnostic insights. Lastly, embedding explainability techniques such as attention maps or saliency visualization may increase clinicians’ trust in AI-driven diagnostic tools and promote clinical integration. In summary, KidneyNeXt demonstrates strong potential as a decision support tool in the diagnostic workflow for kidney tumors. With further validation on larger, real-world datasets and integration into clinical settings, the model could contribute meaningfully to early detection and treatment planning in nephrology and oncologic radiology.

## Figures and Tables

**Table 1:** Details of the proposed KidneyNeXt.

Layer	Operation	Input	Output
Stem	Two parallel conv layers (4 × 4), BN, GELU	224 × 224	56 × 56 × 96
KidneyNeXt 1	3 × 3MaxPool, 3 × 3AvgPool, GroupConv(×2), BN, GELU, 1×	56 × 56 × 96	28 × 28 × 192
KidneyNeXt 2	3 × 3MaxPool, 3 × 3AvgPool, GroupConv(×2), BN, GELU, 1×	28 × 28 × 192	14 × 14 × 384
KidneyNeXt 3	3 × 3MaxPool, 3 × 3AvgPool, GroupConv(×2), BN, GELU, 1×	14 × 14 × 384	7 × 7 × 768
KidneyNeXt 4	3 × 3MaxPool, 3 × 3AvgPool, GroupConv(×2), BN, GELU, 1×	7 × 7 × 768	1 × 1 × 768
Output	Global Average Pooling, FC, Softmax, Classification	1 × 1 × 768	Number of classes

**Table 2:** Distribution of training and test images across classes in the Collected Dataset.

Class	Train Image Count	Test Image Count
Benign	1535	384
Control	906	227
Malignant	918	229

**Table 3:** Class-wise and overall performance metrics of the KidneyNeXt model on the Collected Dataset test set.

	TP	TN	FP	FN	Precision (%)	Recall (%)	F1 Score (%)	Accuracy (%)
**Benign**	384	456	0	0	100	100	100	100
**Control**	225	613	0	2	100	99.12	99.56	99.76
**Malignant**	229	609	2	0	99.13	100	99.57	99.76
**Overall**					99.71	99.71	99.71	99.76

**Table 4:** Distribution of training and test images across classes in the Kaggle CT KIDNEY Dataset.

Class	Train Image Count	Test Image Count
**Cyst**	3557	1332
**Normal**	4875	1828
**Stone**	1329	502
**Tumor**	2187	818

**Table 5:** Class-wise and overall performance metrics of the KidneyNeXt model on the Kaggle CT KIDNEY Dataset test set.

	TP	TN	FP	FN	Precision (%)	Recall (%)	F1 Score (%)	Accuracy (%)
**Cyst**	1331	3147	1	1	99.92	99.92	99.92	99.96
**Normal**	1828	2652	0	0	100	100	100	100
**Stone**	501	3978	0	1	100	99.8	99.9	99.98
**Tumor**	818	3661	1	0	99.88	100	99.94	99.98
**Overall**					99.95	99.93	99.94	99.96

**Table 6:** Distribution of training and test images across classes in the KAUH: Jordan Dataset.

Class	Train Image Count	Test Image Count
Benign	2128	532
Cyst	1064	266
Malignant	1232	308
Normal	1792	448

**Table 7:** Class-wise and overall performance metrics of the KidneyNeXt model on the KAUH: Jordan Dataset test set.

	TP	TN	FP	FN	Precision (%)	Recall (%)	F1 Score (%)	Accuracy (%)
**Benign**	530	1020	2	2	99.62	99.62	99.62	99.74
**Cyst**	265	1286	2	1	99.25	99.62	99.44	99.81
**Malignant**	307	1246	0	1	100	99.68	99.84	99.94
**Normal**	448	1106	0	0	100	100	100	100
**Overall**					99.72	99.73	99.72	99.74

**Table 8:** State of the art studies on kidney CT image classification using deep learning approaches, detailing model architectures, number of classes, dataset sizes, limitations, and key performance metrics.

Study	Methodology	Number of Samples	Limitation	Results (%)
Islam et al. (2022) [27]	Swin Transformer, VGG16, CCT, ResNet, InceptionV3, EANet	Dataset1: 12,446; Dataset2: 9212	Weaker DL models showed poor performance	Dataset1-Swin: Acc 99.30%, Prec 99.30–99.60%, Rec 98.10–100.00%, F1 98.50–99.60%; Densenet201+RF: Acc 99.44%. Dataset2-Swin: Acc 99.52%, VGG16: 97.15%
Alzu’bi et al. (2022) [28]	2D CNN (6-layer), ResNet50, VGG16	4800 CT images (210 patients)	Low VGG16 performance	CNN-6: 97.00%, ResNet50: 96.00%, VGG16: 60.00%
Khan et al. (2025) [35]	ConvLSTM + Inception + Fusion	Dataset1: 2886; Dataset2: 12,446	Class imbalance; no direct limitation specified	Dataset1-Acc 99.30%, Prec 98.00%, Rec 100.00%, F1 99.00%. Dataset2-Acc 91.31%, Prec 69–100%, Rec 75–100%, F1 76–96%
Loganathan et al. (2025) [36]	EACWNet (CNN + SA-CAM)	12,446 (5077 Normal, 3709 Cyst, 2283 Tumor, 1377 Stone)	Low precision in stone class; inter-class variation	Acc 98.87%, Prec 98.25%, Rec 98.71%, F1 98.48%
Rehman et al. (2025) [37]	Swin ViT + DeepLabV3+ + TL	12,446 (5077 Normal, 3709 Cyst, 2283 Tumor, 1377 Stone)	High time/memory cost in some models; ground-truth absence	Acc 99.20%, F1 99.50%, Prec 99.10%, Spec 99.30%
Prabhu et al. (2025) [38]	ProsGradNet (CNN + CGSCR + Context Block)	Prostate: 11,684 train/2854 test/2939 val; KMC: 3432 train/506 test/503 val	High inference time; needs optimization	Prostate-Acc 92.88%, F1 92.92%, Prec 92.91%, Rec 92.93%. KMC-Acc 92.68%, F1 92.63%, Prec 92.76%, Rec 92.73%
Yan et al. (2025) [39]	LRCTNet (LightFire + ResLightFire + Swin distillation)	3090 from 318 patients	Single-center data; poor T1-T2 differentiation; CT only	Acc 95.79%, Prec 93.91%, Rec 93.48%, F1 93.70%, MCC 94.38%
Ayogu et al. (2025) [40]	Ensembles: InceptionV3, CCT, SwinT + VGG16, EANet, ResNet50	12,446 (5077 Normal, 3709 Cyst, 2283 Tumor, 1377 Stone)	Stone class most challenging; low recall in some models	Acc 99.67%, Prec 99.10%, Rec 100.00%
Shanmathi et al. (2025) [41]	Deep Neural Network (DNN)	12,446 (5077 Normal, 3709 Cyst, 2283 Tumor, 1377 Stone)	Only CT-based; no additional biological data	Acc 96.31%, Prec 94.10%, Rec 96.31%
Kashyap et al. (2025) [42]	CNN (custom), ViT (scratch & TL), VGG19, ResNet50	12,446 (Train: 10,955; Test: 1249; Val: 1242)	ViT scratch failed; inter-class similarity; data imbalance	ViT-TL: Acc 99.60%, CNN: 98.16%, ResNet50: 98.48%, VGG19: 94.64%
Kulandaivelu et al. (2025) [43]	AMC-AM (VGG16 + ResNet + Inception) + MSD-CMPA	Not specified in abstract	Poor small-stone detection; weak localization	Acc 95.44%, Prec 95.13%, Rec 95.44%, F1 95.29%, MCC 90.87%
Hossain et al. (2025) [44]	EfficientNet-B7 + ROI + Pixel Reduction	12,446 (5077 Normal, 3709 Cyst, 2283 Tumor, 1377 Stone)	InceptionV3 weak; pixel reduction added limited value	Acc 99.75%, Prec 98.45%, Rec 99.02%, F1 98.78%, AUC 95.78%
Kulkarni et al. (2025) [45]	ResNet-50 + Vision Transformer (Hybrid)	9410 (5915 Normal, 3495 Stone)	Overfitting in some models; data imbalance; explainability emphasized	Acc 99.50%, Loss 2.83% (ResNet+ViT); XResNet 99.36%, MobileNet 98.69%, SwinT 98.13%
Sharon & Anbarasi (2025) [46]	DBAR-Net (attention + dilated CNN)	8750	High class overlap; some class identification difficult	Acc 96.86%, Prec 98.00%, Rec 98.00%, F1 98.00%
Pimpalkar et al. (2025) [47]	Fine-tuned CNNs (VGG16, ResNet50, AlexNet, InceptionV3)	12,446 (5077 Normal, 3709 Cyst, 2283 Tumor, 1377 Stone)	Extremely high accuracy may risk generalizability; high resource demand	InceptionV3: 99.96%, VGG16: 100.00%, ResNet50: 99.85%, AlexNet: 100.00%, CNN: 60.89%
Zain et al. (2025) [48]	CGPCAP (Canny + GLCM + PCA) + CNN classifier	200 (Train: 160, Test: 40)	Small sample size; variable ultrasound quality	Acc 97.50%, Prec 93.75%, Rec 93.75%, F1 93.75%, Spec 98.43%
Chaki & Uçar (2025) [49]	DarkNet19, InceptionV3, ResNet101 + Ensemble + KNN + Bayesian CV	12,446 (5077 Normal, 3709 Cyst, 2283 Tumor, 1377 Stone)	Performance varies by data quality; sample size may be limited	Acc 99.80% (clean), 96.70% (noisy)
Proposed Model	KidneyNeXt	Dataset1: 3199 (Train: 1535/906/918; Test: 384/227/229); Dataset2: 12,446 (Train: 11,948; Test: 1332/1828/502/818); Dataset3: 7770 (Train: 7216; Test: 532/266/308/448)		Dataset1-Acc 99.76%, Prec 99.71%, Rec 99.71%, F1 99.71%. Dataset2-Acc 99.96%, Prec 99.95%, Rec 99.93%, F1 99.94%. Dataset3-Acc 99.74%, Prec 99.72%, Rec 99.73%, F1 99.72%

## Data Availability

The dataset used current study available from the corresponding author on reasonable request.
